# Utility of liquid‐based cytology on residual needle rinses collected from core needle biopsy for lung nodule diagnosis

**DOI:** 10.1002/cam4.3949

**Published:** 2021-05-07

**Authors:** Zhihua Lan, Xiaoli Zhang, Xin Ma, Yiyan Hu, Jing Zhang, Fang Yang

**Affiliations:** ^1^ Department of Pathology The First Affiliated Hospital of University of South China Hengyang China; ^2^ Department of Anorectal Surgery The First Affiliated Hospital of University of South China Hengyang China

**Keywords:** core needle biopsy, cytology of CNB needle rinses, non‐small cell lung cancer, pulmonary nodule

## Abstract

**Background:**

Core needle biopsy (CNB) has become the most common tissue sampling modality for pathological diagnosis of peripheral lung nodules. However, approximately 10% of pulmonary CNB specimens cannot be unambiguously diagnosed, even with auxiliary techniques. This retrospective study investigated the diagnostic value of liquid‐based cytology on residual pulmonary CNB material collected from needle rinses.

**Methods:**

Computed tomography‐guided pulmonary CNB specimens and relevant cytology of CNB needle rinses (CNR) from July 2017 to June 2020 were reviewed. A total of 406 patients, each of whom underwent a CNB procedure, were included in the study.

**Results:**

Of the 406 cases, a more serious diagnosis was rendered by CNR in 6.4% (*n* = 26) of cases. Furthermore, among these 26 cases, 13 malignancies were confirmed only from CNR. Of the remaining 13 patients with uncertain lesions identified from CNR, six were diagnosed with definite benign lesions from tissue samples, five were found to harbor malignant neoplasms through repeated CNB or follow‐up examination, and two had tuberculosis. The sensitivity (320/332, 96.4%) of combined CNR/CNB (both CNR and CNB) in distinguishing malignancies from benign lesions was higher than that of CNB alone (307/332, 92.5%). A total of 320 malignant neoplasms included 198 cases of primary lung adenocarcinoma and 71 cases of primary lung squamous cell carcinoma.

**Conclusions:**

CNR with higher nuclear and cytoplasmic resolution than CNB exhibited a high diagnostic efficacy for differentiating malignant from benign lesions in the lung. Moreover, combined CNR/CNB achieved optimal results in reducing the false‐negative rate and the subtyping of non‐small cell lung cancer.

## Graphical abstract

This retrospective study provides novel insights into a method for evaluating lung nodules in low‐to‐moderate income countries. It elucidates how a combined approach, which utilizes the core needle biopsy (CNB) technique as well as cytology of CNB needle rinses (CNR) containing residual pulmonary CNB material, helps to improve the accuracy and efficacy of diagnosing lung nodules.

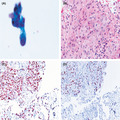

## 1 INTRODUCTION

Small biopsy and cytology are the primary diagnostic modalities used for detecting approximately 70% of advanced lung cancers.^1^ Transthoracic computed tomography (CT)‐guided core needle biopsy (CNB) is widely used as a tissue sampling modality, especially in the peripheral pulmonary nodules, to meet the increasing clinical demand.^2^ Most cases of lung lesions are non‐small cell lung cancer (NSCLC), including adenocarcinoma (ADC) and squamous cell carcinoma (SQC). The 2015 World Health Organization Classification of Lung Tumors guidelines indicate that personalized treatment based on molecular testing requires a more precise histopathological subtyping through immunohistochemistry.^3^ Mutations in epidermal growth factor receptor (*EGFR*) and rearrangements in anaplastic lymphoma kinase (*ALK*) and c‐ros oncogene 1 (*ROS1*) have emerged as effective therapeutic targets for advanced lung adenocarcinoma.^4^

Although CNB has been used frequently in current practice, some cases cannot be definitively diagnosed owing to a sampling error or technical failure. Concerning malignancies, the nonspecific benign findings may lead to repeated biopsy or unnecessary follow‐up evaluation. As another sampling modality, fine needle aspiration (FNA) is still used at some diagnostic centers.^5^ Previous studies have shown that a combined lung FNA/CNB(both FNA and CNB) approach has a high diagnostic efficacy and accuracy for patients with lung mass lesions.^6^ In addition, the cytology of the FNA material provides more evidence on the nucleus and cytoplasm morphology than hematoxylin and eosin‐stained CNB.^7^ However, concurrent use of FNA and CNB involves higher medical costs than when using CNB alone. Therefore, a diagnostic solution is needed to meet the requirements of low cost and high accuracy.

In the past, the needle used in CNB was usually discarded after the procedure was completed. Recently, the needle rinse fluid has emerged as a diagnostic material for detecting microbial infection and specific gene mutations in malignancies.^8^, ^9^ Needle rinse fluid can be easily obtained by flushing the cell preservation liquid through the needle several times after the biopsy tissue is placed in a fixative solution. As an additional material, needle rinse fluid is a rich source of microbial or tumor cell deoxyribonucleic acid (DNA).

Cytology of the transbronchial biopsy needle rinse solution has been previously described.^10^ At our institution, the needle rinse fluids are collected for all CNB cases and sent for cytology. The diagnoses of biopsy tissues and relevant cytological analyses are conducted separately by a respiratory pathologist and a cytologist, respectively. A difficult case, with either an ambiguous biopsy‐based diagnosis or a serious discrepancy between the biopsy and cytology findings, is referred to senior physicians for review.

Therefore, the aims of our study were as follows: (1) To evaluate the sensitivity and specificity of cytology of CNB needle rinses (CNR); (2) to determine the superiority of combined CNR/CNB over CNB; and (3) to explore the discordance between CNB and CNR findings.

## 2 MATERIALS AND METHODS

### 2.1 Patients

The database at our Pathology Department was searched for CNB specimens and relevant CNR dating from July 2017 to June 2020. Eight repeated CNBs were excluded from this study, as the objective was to only test samples of initial CNBs. To determine the diagnostic accuracy, patients with a final diagnosis of benign disease were included. These patients were enrolled only if their diagnoses were confirmed via their surgical specimens, with the lesions either regressing with or without medical treatment (excluding antitumor therapy), or remaining stable in size for at least 6 months.^11^ Additionally, malignant cases primarily diagnosed as benign or uncertain (e.g., atypical cells that could not be identified as benign or malignant) were included if they were confirmed through repeated CNBs or surgical specimens. Therefore, 406 cytologic‐histologic pairs in total were included in our study.

### 2.2 Specimen collection

The CT‐guided CNB procedure was performed by an experienced oncologist with the help of a radiologist in the Department of Interventional Radiology at our institution. We used 18‐gauge side‐cutting core biopsy needles (MISSION^®^, Bard Peripheral Vascular Inc.) to retrieve CNB samples. After the CNB tissue was placed in 10% neutral formaldehyde, 15 ml of cell storage solution (Guangzhou Anbiping Medical Company Technology Co., Ltd.) was flushed through the needle several times to obtain the rinse fluid sample. All the rinse fluid samples were collected and sent for cytology.

### 2.3 Specimen processing

Using the Sedimentation Cell Prep Plus LBC (liquid‐based cytology) Processor under the liquid‐based preparation (LBP) system (LBP‐2601, Guangzhou Anbiping Medical Company Technology Co., Ltd.), cells were automatically sedimented onto a glass slide, forming a diagnostic area of 13 mm in diameter.^12^ The area was stained using Papanicolaou stain. The CNB tissue was embedded in paraffin, sliced into 3‐μm‐thick sections, and stained with hematoxylin and eosin.

The CNR samples were evaluated by the same cytopathologist blinded to the biopsy‐based diagnosis. The NSCLC samples also underwent immunohistochemical analysis, which helped verify the diagnosis. P63, P40, and cytokeratin 5/6(CK5/6) were markers for SQC, and cytokeratin 7(CK7), NapsinA, and thyroid transcription factor‐1(TTF‐1) were used to identify ADC. All six antibodies were purchased from Maixin Biotechnology. An experienced pathologist, specializing in both cytopathology and respiratory pathology, made a final comprehensive diagnosis based on observations from all the various staining procedures performed.

### 2.4 Statistical analysis

We categorized the original cytological and pathological results into three groups: (1) positive (malignancy or favor of malignancy); (2) uncertain (a few cells could not be identified as benign or malignant); and (3) negative (no malignant cells were found).

The sensitivity, specificity, accuracy, and false‐negative rate of CNR, CNB, and combined CNR/CNB for the diagnosis of malignancy were determined from the follow‐up data. The *χ* ^2^ test was performed to assess the differences between CNR and CNB as well as between CNB and combined CNR/CNB. Results were considered statistically significant at *p* ≤ 0.05. The ability of NSCLC subtyping by CNR was also reviewed.

## 3 RESULTS

As presented in Table 1, the distribution of patients into the positive, negative, and uncertain categories was significantly different for CNB (75.6%, 21.4%, and 3.0%, respectively) and CNR (57.1%, 28.1%, and 14.8%, respectively) (*p* < 0.001). Of the 406 paired CNR/CNB specimens, 320 (78.8%) were diagnosed as malignancies, 73 (18.2%) as benign lesions, and 12 (3.0%) as uncertain through the combined method. The distribution of patients into the positive, negative, and uncertain categories was similar for combined CNR/CNB and CNB (*p* = 0.416).

**TABLE 1:** Distribution of cases assessed by CNR, CNB, or combined CNR/CNB into categories reflecting the property of the lesions (*N* [%])

Result	CNR	CNB	CNR/CNB
Positive	232 (57.1%)	307 (75.6%)	320 (78.8%)
Negative	114 (28.1%)	87 (21.4%)	74 (18.2%)
Uncertain	60 (14.8%)	12 (3.0%)	12 (3.0%)
Total	406	406	406

Abbreviations: CNB, core needle biopsy; CNR, cytology of needle rinses; CNR/CNB, diagnosis based on the combination of CNB and CNR; Negative, negative for malignancy; Positive, positive for malignancy; Uncertain, uncertain cells that could not be identified as benign or malignant.

As illustrated in Table 2, a more serious diagnosis was made using CNR in 26 (26/406, 6.4%) cases. These cases were distributed as follows: positive CNR/negative CNB (*n* = 6), positive CNR/uncertain CNB (*n* = 7), and uncertain CNR/negative CNB (*n* = 13). The six cases with positive CNR/negative CNB comprised three ADCs and three SQCs. The two ADCs were verified by repeated CNBs and the other one was proved by EGFR exon 21 p.L858R mutation detected in blood specimen. The three SQCs demonstrated sufficient cells with abnormal keratinization. Therefore, subsequent pathological examinations were not performed. The seven cases of positive CNR/uncertain CNB included six primary ADCs and one metastatic ADC from the stomach. Four of the primary ADCs were verified by repeated CNB, one was confirmed by the detection of adenocarcinoma cells in pleural effusion, and the other one was finally confirmed by detection of a few CK7‐positive atypical cells combined with typical morphological features on CNR. Six of the 13 (46.1%) patients with uncertain CNR/negative CNB outcomes were confirmed as having specific benign lesions through CNB. The other seven patients were finally shown to include five malignancies and two tuberculosis cases by follow‐up examination and therapy.

**TABLE 2:** Correlation between CNR and CNB based on the distinguished categories of lesions

	CNR			Total
	Positive	Uncertain	Negative	
CNB				
Positive	219	43	45	307
Uncertain	7	4	1	12
Negative	6	13	68	87
Total	232	60	114	406

Abbreviations: CNB, core needle biopsy; CNR, cytology of needle rinses; Negative, negative for malignancy; Positive, positive for malignancy; Uncertain, uncertain cells that could not be identified as benign or malignant.

One of the four patients with uncertain CNR/uncertain CNB results was confirmed as tuberculosis. The other three included two primary ADCs proved separately by CNB and pleural effusion examination, and one metastatic renal clear cell carcinoma verified by FNA on renal mass. The one case with negative CNR/uncertain CNB outcome was verified as bronchiectasis in surgically excised specimen.

**FIGURE 1:**
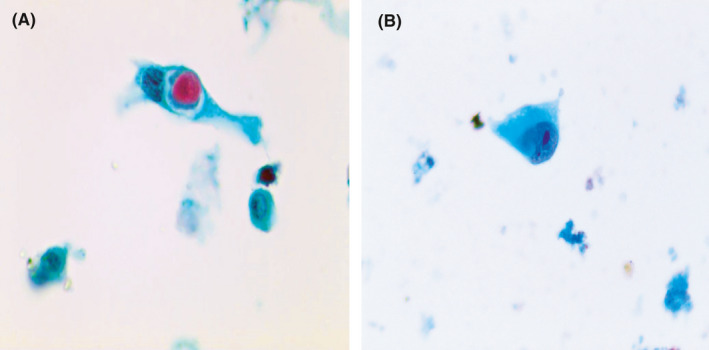
(A) Definite keratinization and coarse chromatin fibers favoring squamous cell carcinoma (Papanicolaou stain, 400×). (B) Neoplastic cells with prominent nucleoli and flat luminal edges suggesting adenocarcinoma (Papanicolaou stain, 400×)

**FIGURE 2:**
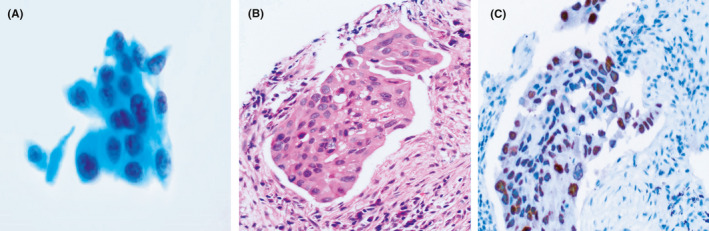
(A) Rough nuclear chromatin and dense cytoplasm seeming to reveal squamous cell carcinoma (Papanicolaou stain, 400×). (B) Pink cytoplasm and sharp cell borders mimicking squamous cell carcinoma (hematoxylin and eosin staining, 200×). (C) Positive TTF‐1 supporting adenocarcinoma (Envision, 200×)

**FIGURE 3:**
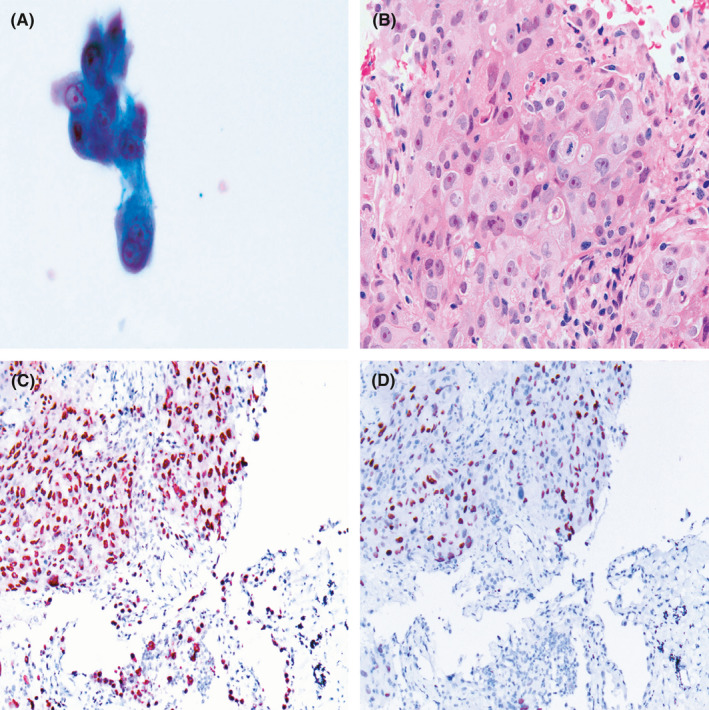
(A) Thickened nuclear membrane and large nucleolus favoring adenocarcinoma (Papanicolaou stain, 400×). (B) Tumor cells arranged in a solid pattern exhibiting squamous differentiation (hematoxylin and eosin staining, 200×). (C) Tumor cells positive for TTF‐1 (Envision, 200×). (D) Tumor cells locally positive for P63 (Envision, 200×)

The combined CNR/CNB findings are summarized in Table 3. Of the 320 malignant cases diagnosed through combined CNR/CNB, 311 (97.2%) cases were identified as primary neoplasms and 9 (2.8%) were metastatic or recurrent malignancies. Metastatic neoplasms comprised seven cases of ADC (breast, *n* = 3; liver, *n* = 1; stomach, *n* = 1; and colon, *n* = 2) and one malignant solitary fibrous tumor from the right thigh. The recurrent case was diffuse large B‐cell lymphoma, primarily localized in the stomach. Primary malignancies included 198 cases of ADC, 71 cases of SQC, and 15 cases of small cell lung carcinoma (SCLC). Malignant lesions were accurately differentiated from benign lesions through CNR alone in 232 of 320 (72.5%) cases. Using CNR alone, 114 (114/198, 57.6%) cases of ADC, 28 (28/71, 39.4%) cases of SQC, and 8 (8/15, 53.3%) cases of SCLC were diagnosed (Figure 1). However, in four cases, SQC was misdiagnosed as ADC, and in 12 cases, ADC was misdiagnosed as SQC through CNR (Figure 2). Furthermore, in seven cases, adenosquamous carcinoma was initially diagnosed as ADC, SQC, or NSCLC through CNR alone (Figure 3).

**TABLE 3:** Summary of the results of combined CNR/CNB and subtyping of neoplastic cells

	CNR						
	ADC	SQC	SCC	NSCLC	Negative	Uncertain	Total
CNR/CNB							
ADC	114	12	1	20	25	26	198
SQC	4	28	1	14	13	11	71
SCC	1	0	8	0	5	1	15
NSCLC	1	0	0	5	1	2	9
Negative	0	0	0	0	67	6	73
Uncertain	0	0	0	0	1	11	12
LCNEC	1	0	1	2	0	0	4
ASC	3	3	0	1	0	0	7
ADCC	0	0	0	0	0	1	1
MEC	1	0	0	0	1	0	2
MM	0	0	0	1	0	1	2
DLBCL	0	1	0	0	0	0	1
SFT	1	0	0	1^a^	0	0	2
LMS	0	0	0	1	0	0	1
Metastatic SS	0	0	0	0	0	1	1
Metastatic ADC	6	0	0	1	0	0	7
Total	132	45	11	45	113	60	406

Abbreviations: ADC, adenocarcinoma; ADCC, adenoid cystic carcinoma; ASC, adenosquamous carcinoma; CNB, core needle biopsy; CNR, cytology of needle rinses; CNR/CNB, diagnosis based on the combination of CNB and CNR; DLBCL, diffuse large B‐cell lymphoma; LCNEC, large cell neuroendocrine carcinoma; LMS, leiomyosarcoma; MEC, mucoepidermoid carcinoma; MM, malignant mesothelioma; Negative, negative for malignancy; NSCLC, non‐small cell lung carcinoma; SCLC, small cell lung carcinoma; SFT, solitary fibrous tumor; SQC, squamous cell carcinoma; SS, synoviosarcoma; Uncertain, uncertain cells that could not be classified as benign or malignant.

^a^ Metastatic malignant solitary fibrous tumor from the right thigh.

**FIGURE 4:**
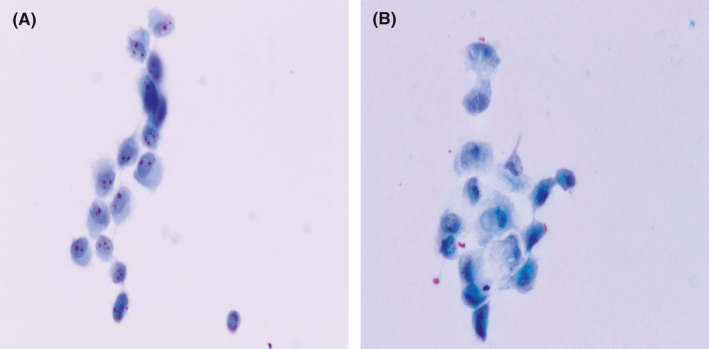
(A) Atypical cells with inconspicuous nucleoli speculated as neoplastic alveolar epithelium, despite a negative CNB result (Papanicolaou stain, 400×). (B) Uncertain cells proven to be degenerated epithelioidcells by CNB (Papanicolaou stain, 400×)

Of the 307 malignancies diagnosed through CNB, negative outcomes were obtained in 45 cases, and an uncertain diagnosis was obtained in 43 cases through CNR. The main reason for the uncertain CNR diagnosis was low cellularity. Furthermore, necrotic material intervened in the observation and hindered a definite diagnoses. Atypical cells were classified as uncertain lesions in CNR, not only because they were insufficient in number but also because some tumor cells showed lesser atypia than other cells (Figure 4A). Additionally, some benign cells could mimic malignant cells (Figure 4B). The uncertain cells were recognized in CNB as follows: neoplastic cells from myelolipoma in one case, myofibroblasts in two cases, and epithelioid cells in three cases.

Follow‐up evaluation revealed 332 malignancies. The sensitivities of combined CNR/CNB and CNB alone for the diagnosis of malignancy were 96.4% (320/332) and 92.5% (307/332), respectively. Both procedures presented 100% specificity. The 12 malignancies without a definite diagnosis through combined CNR/CNB were distributed as follows (Table 4): uncertain CNR/uncertain CNB (*n* = 3), uncertain CNR/negative CNB (*n* = 5), and negative CNR/negative CNB (*n* = 4). The accuracies of combined CNR/CNB and CNB alone were 97.0% (394/406) and 93.8% (381/406), respectively. The calculated false‐negative rates were 3.6% and 7.5% for combined CNR/CNB and CNB alone, respectively.

**TABLE 4:** Final diagnoses for patients with negative and/or uncertain CNR/CNB results

CNR/CNB	Malignant (*n*)	Benign (*n*)	Total (*n*)
Uncertain/negative	5	8	13
Uncertain/uncertain	3	1	4
Negative/negative	4	64	68
Negative/uncertain	0	1	1
Total	12	74	86

Abbreviations: CNB, core needle biopsy; CNR, cytology of needle rinses; CNR/CNB, diagnosis based on the combination of CNB and CNR; Negative, negative for malignancy; Uncertain, uncertain cells that could not be identified as benign or malignant.

## 4 DISCUSSION

The traditional standard sampling modality, CNB, can obtain greater amounts of tissue and display higher sensitivity than FNA.^6^, ^13^ A recent study reported that 116 (85%) radiologists prefer performing CNB. Among them, 57 (42%) used CNB alone and the rest used CNB in combination with FNA.^6^ Although the sensitivity of CNB alone reaches 85.7%–97.4%,^14^ it is necessary to decrease the rate of false‐negative results because repeated CNB increases the risk of complications, and false‐negative results delay the treatment. Therefore, combined FNA/CNB has been encouraged in some studies with the advantage of minimizing sampling errors and compensating for the limitations of other methods.^2^, ^14^ However, concurrent FNA not only indicates extra cost, but also influences the diagnostic yields of CNB being performed after FNA.^14^ Additionally, some institutions, especially in developing countries, lack sufficient facilities and cytologists specializing in rapid on‐site evaluation compared with the institutions in developed countries.^15^ Therefore, such institutions cannot perform CNBs with sensitivities similar to or higher than that of institutions in developed countries, although no statistical data have been reported. Touch preparations for rapid on‐site evaluation of CNBs are also considered cytological diagnostic materials.^16^ However, inappropriate touch preparations may limit the potential for molecular profiling.^17^

Utilization of needle rinse fluid in combination with CNB was found to be the best strategy when compared with the high medical costs of combined FNA/CNB and CNB alone with about 10% false‐negative rate, even though combined FNA/CNB has higher accuracy than CNB alone. CNR correctly recognized 69.9% of the malignancies. The cytology of transbronchial needle aspiration rinse fluid revealed a positive result for only 25% of the primary malignancies.^10^ The reason behind the high detection rate in our study was that all the CNR samples were submitted for cytological examination as opposed to only one out of the four transbronchial needle aspiration rinse fluid samples being sent for the same examination in the previous study. Another reason may be associated with the low cell loss rate of the sedimentation technique.^12^

Histological specimens of CNB exhibit formalin‐fixation artifacts; on the contrary, CNR reveals morphological characteristics of neoplastic cells with greater resolution than the former owing to better preservation techniques.^7^, ^18^ Another advantage of CNR is that it serves as a remedial procedure when encountering failure with CNB. Overall, 3.2% (13/406) of the cases in our study were diagnosed as malignant through CNR alone. Finally, cases with discordant CNR diagnoses promoted the understanding of the cytology.

CNR was useful in not only determining whether the tumor was benign or malignant, but also distinguishing the exact NSCLC subtype. Orange G, a component of the Papanicolaou stain, highlights keratinized cells; therefore, it is convenient to observe keratinized SQC.^19^ The three‐dimensional arrangement of the ADC could be visualized in CNR owing to the sedimentation technique. In general, ADC cells are arranged in gland‐like or papillary patterns. However, the SQC cells exhibited solid or sheet‐like structures. Accurate morphologic distinction of ADC and SQC is difficult in poorly differentiated NSCLC at the end of the spectrum because of overlapping architectures and cytological features.^20^ In clinical practice, extremely precise adherence to one or two diagnostic features is not advisable. For example, large prominent nucleoli, one of the most conspicuous signs of ADC, may also be observed in non‐keratinizing SQCs. Moreover, neoplastic cells in early lung ADC may display inconspicuous nucleoli. The morphological diversity of ADC is not only in terms of the size of the nucleoli, but also in terms of cell size, chromatin, and nucleo‐cytoplasmic ratio. In general, more accentuated cytoplasmic density, more apparent coarseness, and inconspicuous nucleoli favor poorly differentiated non‐keratinizing SQCs, whereas the presence of intracellular mucin droplets is suggestive of ADC.^21^

However, if a cytologist reluctantly makes a diagnosis of malignancy based on an insufficient amount of cells, the positivity may be increased at the risk of a decrease in specificity. Moreover, the accuracy of the tumor sub‐classification may be impaired.

It has been reported that a cytological diagnosis of SCLC is reliable.^21^ However, one case of SCLC in our study was misdiagnosed because the cytology was accidentally speculated to be a well‐differentiated lung ADC. Moreover, NSCLC was an approximate diagnosis when the morphology was found to be ambiguous in CNR. Additionally, CNB can yield more accurate results based on ancillary immunohistochemistry and special staining, which cannot be used on scant CNR materials. Finally, even in terms of visualization of morphological features, CNR did not demonstrate an overall superiority over CNB, as interpreted from the following observations: First, the hematoxylin and eosin‐stained CNB specimens displayed intercellular bridges more clearly than the CNR specimens. Second, myofibroblasts, macrophages, epithelioid cells, benign neoplastic cells, and especially malignant cells may be suspected to be atypical owing to morphologic mimicry and a lack of adjacent stromal cells. Third, neoplastic cells of well‐differentiated ADCs appear similar to proliferative alveoli epithelial cells in CNR, thereby possibly confounding cytologists. Fourth, crush artifacts, which may have resulted from the pressure between the needle and tissue, occurred in some aggregated cells. Therefore, the availability of CNR results before the tissue biopsy reports plays an auxiliary role in the final diagnosis with combined CNR/CNB. Strict diagnostic criteria were applied in CNR to preserve accuracy and avoid false‐positive results, even if some malignancies were sub‐classified as uncertain lesions.

Previous studies have attributed most false‐negative results in CNB to sampling errors or technical failure.^22^ In this study, of the 11 malignant cases with negative CNB results, 6 were verified and 5 were suspected through CNR alone. Of the 10 malignant cases with uncertain CNB results, seven were confirmed and three were uncertain through CNR alone. Therefore, we speculated that tissue fixation and tissue block trimming impaired the yield of malignant cells and accounted for most of the false‐negative results.

Management of patients with uncertain CNR/CNB results is essential for advanced therapy. Patients (3/4, 75%) with uncertain CNR/uncertain CNB results were subsequently diagnosed with a high risk of malignancy. The second highest risk was observed in five out of seven cases with uncertain CNR/negative CNB results (5/7, 71.4%). The other 6 cases with uncertain CNR diagnoses were not considered for calculating the risk rate because of confirmation of benign disease by CNB. One case with a negative CNR/uncertain CNB result was finally confirmed as nonspecific inflammation through surgical specimens. Owing to the scarcity of cases, the risk rate associated with this category was difficult to evaluate. Additionally, 4 out of 68 cases (5.9%) with negative diagnoses through both methods were finally verified as malignant lesions. Viewed in a certain light, the false‐negative rate of combined CNR/CNB was remarkably lower (4/332, 1.2%). Irrespective of the manner of data collection and interpretation, the most important aspect is planning appropriate treatment strategies based on the calculated risk. Moreover, the strategy used for negative results was vital. Specific benign lesions were treated with etiological therapy. Nonspecific benign lesions were reviewed by a multidisciplinary team. False‐negative results for non‐cancerous lesions also had a negative influence on the diagnosis. For instance, a patient with fungal infections was not definitively diagnosed until the surgical specimen was examined.

Our study is mainly limited by its retrospective design, which could not be randomized. Some patients lost to follow‐up were not enrolled in the study, which resulted in a selection bias. Furthermore, cytologists might tend to subconsciously diagnose ADC or favor ADC when cells with equivocal features are encountered. There may have been an observation bias, which cannot be exactly estimated. Lastly, we did not review molecular tests, which were completed in approximately 10% of the paraffin‐embedded CNB tissue specimens at our institution.

## 5 CONCLUSIONS

CNR can help recognize approximately 70% of malignancies, nearly half of which are sub‐classified based on morphology. Therefore, a combined CNR and CNB procedure in patients is the best method for evaluating lung nodules in low‐to‐moderate income countries.

## CONFLICT OF INTEREST

The authors declare no potential conflicts of interest.

## ETHICAL APPROVAL STATEMENT

This retrospective study was approved by the Research Ethics Board at the First Affiliated Hospital of University of South China. The need for informed consent was waived by the Research Ethics Board.

## Data Availability

The data that support the findings of this study are available from the corresponding author upon reasonable request.
